# Feasibility of using a biofeedback device in mindfulness training - a pilot randomized controlled trial

**DOI:** 10.1186/s40814-021-00807-1

**Published:** 2021-03-24

**Authors:** Brenna Lin, Christopher Prickett, Steven Woltering

**Affiliations:** Department of Educational Psychology, Texas A&M University, 718B Harrington Tower, TAMU, College Station, TX 77843-4225 USA

**Keywords:** Meditation, Biofeedback, Stress, College students, Randomized controlled trial, Training

## Abstract

**Background:**

Stress can negatively impact an individual’s health and well-being and high levels of stress are noted to exist among college students today. While traditional treatment methods are plagued with stigma and transfer problems, newly developed wearable biofeedback devices may offer unexplored possibilities. Although these products are becoming commonplace and inexpensive, scientific evidence of the effectiveness of these products is scarce and their feasibility within research contexts are relatively unexplored. Conversely, companies are not required, and possibly reluctant, to release information on the efficacy of these products against their claims. Thus, in the present pilot, we assess the feasibility of using a real-time respiratory-based biofeedback device in preparation for a larger study. Our main aims were to assess device-adherence and collaboration with the company that develops and sells the device.

**Method:**

Data were collected from 39 college students who self-identified as experiencing chronic stress at a Southwestern university in the USA. Students were randomized into either a mindfulness-only control group without a biofeedback device (*n* = 21), or an experimental group with biofeedback device (*n* = 18). Both groups received mindfulness meditation training. Pre-test and post-test procedures were conducted 2 weeks apart. Further, both participant compliance and company compliance were assessed and collaboration with the company was evaluated.

**Results:**

Participant device-adherence as well as the company’s collaboration necessary for a full-scale study was determined to be low. This may also have affected our results which showed a strong main effect for time for all outcome variables, suggesting all groups showed improvement in their levels of stress after the intervention period. No group by time effects were identified, however, indicating no added benefit of the biofeedback device.

**Conclusions:**

Our findings suggest feasibility of future studies requires full collaboration and detailed and agreed upon data sharing procedures with the biofeedback company. The particular device under investigation added no value to the intervention outcomes and it was not feasible to continue a larger-scale study. Further, as the technology sector is innovating faster than it can validate products, we urge for open science collaborations between public and private sectors to properly develop evidence-based regulations that can withstand technological innovation while maintaining product quality, safety, and effectiveness.

**Trial registration:**

NCT02837016. Registered 19 July 2016.

**Supplementary Information:**

The online version contains supplementary material available at 10.1186/s40814-021-00807-1.

## Background

### Stress and barriers to treatment

Mental health problems related to stress and anxiety are an increasingly prevalent issue facing college students today [1–7]. This is an alarming trend considering the host of negative associations related to stress and anxiety such as decreased academic performance1 [8–10], school satisfaction [11], depression [5, 6, 12], health problems [13, 14], substance use [5, 15–18], poor sleep [19], unhealthy eating behavior [18, 20], and reduced self-efficacy [21, 22]. With this in mind, stress is an important target for prevention and treatment on college campuses. Despite the general effectiveness of cognitive behavioral therapy (CBT) [23–28], there are problems reducing its ultimate usefulness as a treatment for some people related to a lack of help-seeking behavior due to stigma, time-constraints, privacy concerns, and costs [29–31]. Furthermore, a problem that traditional treatment faces is the difficulty individuals experience applying the techniques learned in therapy to real life emotionally charged problems. This has also been dubbed the transfer problem [32]. Notably, stress and anxiety may be particularly susceptible to the transfer problem as patients often receive interventions in calm, controlled settings removed from distressing events or triggers that may lead to stressful thoughts or feelings. Once faced with these stressors, however, they may find it difficult to apply what was learned in therapy in these moments when they lack situational control. Furthermore, the emotions of fear that grip people in stressful states is characterized by reduced cognitive control and a rigid fixation on perceived concerns [33–35] making it even more challenging to break loose from habitual and unhelpful thought patterns.

With the above in mind, it is important to consider and develop treatment options that can effectively reduce stress and anxiety while also circumventing the many barriers that reduce the usefulness of traditional treatment related to help-seeking and transfer. The primary aim of the present pilot was to assess the feasibility of testing a low-cost wearable biofeedback device based on mindfulness medication techniques in college students with high levels of self-reported stress.

### Meditation, mindfulness, and biofeedback devices as possible solutions

Meditation interventions may address some of the shortcomings of traditional treatment. For instance, an array of resources exists for meditation, including online videos, apps, and tutorials, addressing the cost and privacy barriers that exist with traditional treatments. Additionally, meditation is often associated with a lifestyle and may not carry the stigma of being in therapy which may make it easier for individuals to seek treatment. Furthermore, self-reported improvements in stress levels have been indicated in as little as 30 min [36, 37] reducing the time and commitment needed in order for individuals to feel and see improvement. Notably, evidence for the effectiveness of meditation is increasing. Meditation based practices and trainings have been shown to improve individual’s abilities to regulate attention [38], improve executive functioning [38, 39], improve emotion regulation [40], and strengthen neural systems needed for emotion regulation [41]. In relation to stress, a number of studies have found that regular mindfulness practices can reduce stress [42–44].

Mindfulness, a form of meditation, is characterized as a way of paying attention in a purposeful way [42]. Intentionally directing one’s attention can be regarded as a self-regulatory practice and is often achieved through meditation [45]. While mindfulness meditation can refer to many practices, the core of these practices is the moment-to-moment awareness of one’s body and mind, without judgment or criticism. This is often done by focusing attention on one’s own breath, promoting cognitive control [46]. Focusing one’s attention on the present moment can reduce negative rumination and worry about the future, common in highly stressed individuals.

The attentional focus on breathing slowly and in a regulated fashion may help reduce stress as breathing has significant influence on self-regulatory processes such as cardiac modulation [47]. Individuals under high stress consistently exhibit erratic breathing patterns, while individuals under low stress are shown to exhibit slow, regulated breathing patterns [48, 49]. The relationship between the breath and cardiac modulation is bi-directional, in that changes in breath rate can also influence stress levels [50, 51]. Moreover, alterations in respiratory patterns are proven to affect an individual’s physiological and emotional arousal as well as perceptions of negative distress [50, 52, 53], while short-term modification of breathing patterns has been found to reduce the subjective experience of stress [54]. Solutions to the transfer problem, however, are more difficult to find or to practically realize. One would have to have therapeutic settings closely resemble or simulate the real-life situations that patients experience their problems in. This appears hard and costly to do but would, if realized, likely increase the efficacy of treatment. Indeed, the benefits of treatment are often the largest when such an approach is possible.

Various newly developed wearable biofeedback devices, however, may offer unexplored possibilities. Biofeedback involves observing one’s own physiological responses and modulating behavioral patterns in real time [55], possibly providing a solution to combat the transfer problem encountered by many health providers. Wearable biofeedback devices can deliver prompts based on continuously monitored physiological signals. Prompts, such as minor vibrations, text messages, or other cues, can provide real-time feedback cueing wearers to adjust unhealthy habits. These devices can remind wearers of their mental and physiological state and signal them to perform behavioral cues in real-life contexts *at the exact moments* they are actually experiencing certain emotions. Specifically, a signal to remind individuals to modulate their respiratory patterns may increase the transfer of learning. Wearable devices also have the advantage that they are becoming increasingly more affordable and available. Self- tracking of health factors such as weight, exercise, blood pressure, sleep, and blood sugar are now commonplace and can be provided at relatively low-cost [56].

### Public-private disconnect

Furthermore, biofeedback interventions include modulation and direction of awareness towards one’s own physiology, akin to the process of mindfulness. Through feedback signals from the device, individuals are able to gain increased awareness of physiological processes and ideally gain control and modulate these processes. The use of biofeedback-aided interventions for stress and anxiety has proven successful with various populations including school aged children [43], college students [57], professionals [58], athletes [59], and individuals suffering from mental illnesses, (e.g., depression and post-traumatic stress disorder) [60–62]. A majority of evidence, however, utilizes non-portable biofeedback devices, which limits the ecological validity. Furthermore, non-portable biofeedback devices are costly and access to these devices is often restricted, requiring permission through medical offices or wellness centers. Hence, considering the limited literature on this topic, there is a clear need for more robust discussions on the feasibility of using these biofeedback devices within the context of randomized control trials (RCT).

In our current day and age, private companies are not required to provide detailed information regarding feasibility and effectiveness of released products, unless they are seeking FDA approval. While these devices are relatively inexpensive for individual users, the overall wearable market is growing and predicted to increase over the next few years, with global estimates ranging between $25 and $44 billion dollars, by 2020 [63–65]. The exponential growth of technology [66] has produced an ecosystem of relatively inexpensive technological products that may or may not provide real clinical value. Currently, there is a lack of incentivization for private companies to partner with researchers. Further, these health-related technological devices do not require clinical trials, to assess the efficacy, prior to making claims. Despite the large market for this industry, to the best of our knowledge, literature supporting the use of wearable biofeedback devices is often theoretical [67] or mechanical [68, 69] in nature, while applied research of products is scarce (despite a few studies, see [70, 71]). Moreover, we could not identify literature pairing wearable biofeedback devices with meditation as a treatment option. Coupling the lack of literature with the vast number of devices that exist on the market indicates a need to test the feasibility of these devices as well as evaluate the quality of participant adherence and collaboration with private companies.

### The current study

The literature suggests a strong potential for meditation and biofeedback treatments in reducing stress [72, 73]. Further, because of mindfulness meditation’s focus on respiration and physiology, the integration of respiratory-based biofeedback and mindfulness meditation appears evident. Our study had two key objectives. Our primary objective related to feasibility and had two key components. First, we needed to assess adherence with the device. We will test this using the amount of missing data due to adherence. Second, we needed to assess the biofeedback company’s communication and transparency. This was important because if we showed the device was effective, we would need to determine the mechanism of action in a larger RCT. This objective could be tested by our research team gaining access to the algorithms and raw data. Our expectation was that both the participants and the biofeedback company with comply with our study design and data requirements in such as it would be feasible to run a larger scale study.

A secondary objective was to estimate the effectiveness of a wearable biofeedback device in addition to the effects of a mindfulness meditation intervention. This was important because if the device did not demonstrate any potential effectiveness, we may have to seek a different type of biofeedback device or question our theoretical assumptions. If there was evidence that the device conferred added benefit and that data feasibility was acceptable, we would design a larger and more elaborate study with a waitlist control group that would confirm these findings and also test more specifically for the mechanisms of improvement. We note that, due to the current experimental design of the pilot, we cannot make claims regarding the effectiveness of the mindfulness treatment nor the mechanism of action. We can only make claims as to the estimated added effectiveness of the specific biofeedback device tested.

## Methods

### Participants

University students who self-identified as experiencing chronic stress were recruited through flyers and a campus wide email on a large Southwestern United States campus between February and July of 2017. Inclusion criteria were as follows: (1) at least at least 18 years of age and be a student at time of testing, (2) self-identify as highly stressed. Exclusion criteria included (1) major psychiatric or developmental disorders besides depression or anxiety, (2) limited proficiency in English language.

### Sample size

**Fig. 1:**
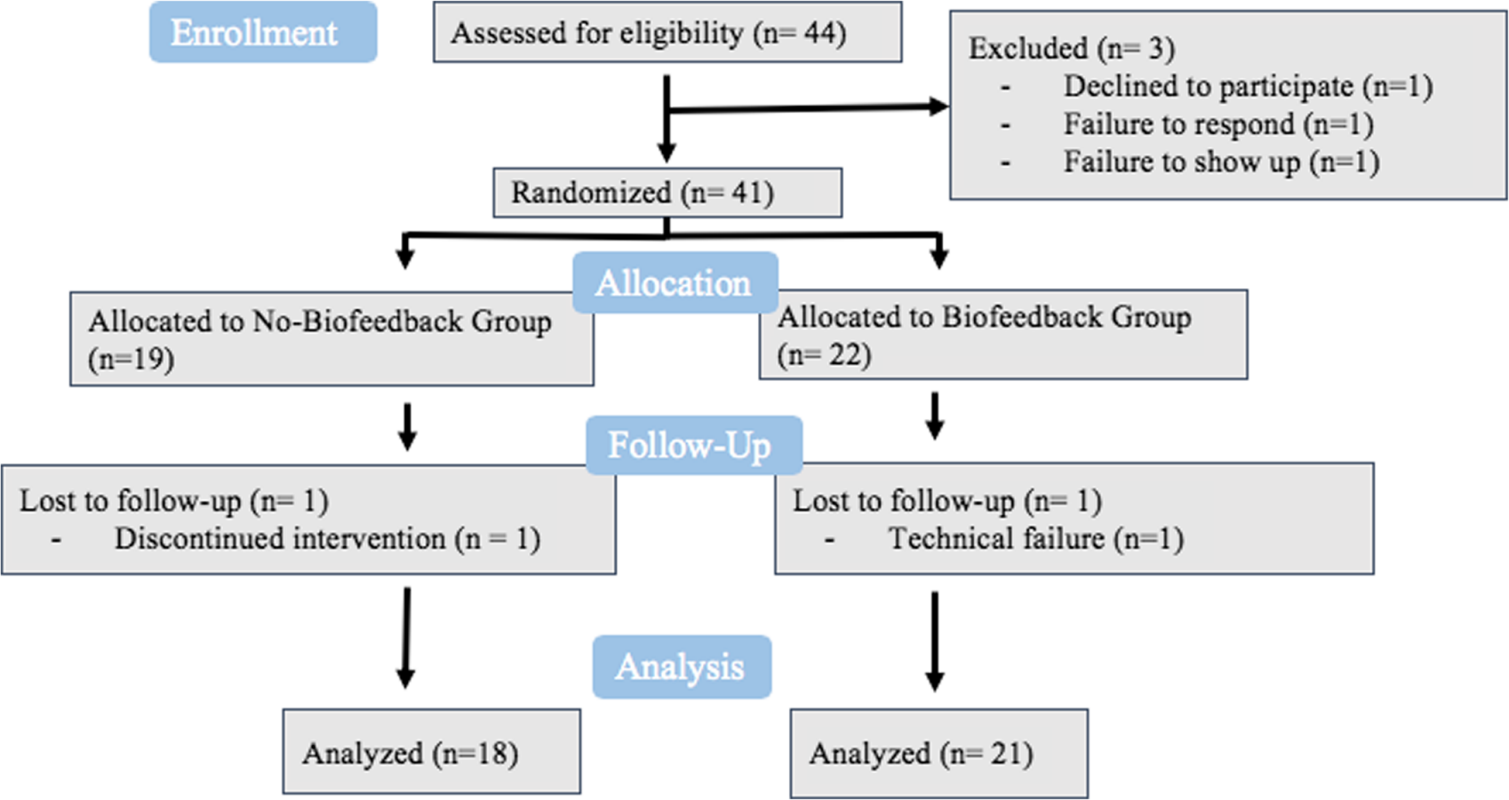
Flow diagram of participants

Figure 1 shows a detailed chart in accordance with the Consolidated Standards of Reporting Trials (CONSORT) reporting standards of the flow of participants through our study. Eligible participants were randomized, before they visited the lab using a simple randomization scheme through the website www.randomization.com, into two arms: a meditation treatment with biofeedback group (the biofeedback experimental group; final *n* = 21) and a meditation without biofeedback group (no-biofeedback control group; final *n* = 18). As no previous studies have been conducted on this topic, no effect size could be identified for power analysis. Additionally, as this was a pilot study, we decided to keep the groups to approximately 20 each.

### Lab procedure

Eligibility screening was conducted via a brief phone call. Following the eligibility screening, participants visited the psychology lab, located on the campus of the university, for their first visit where they signed informed consent before data collection began. First, participants completed a set of questionnaires relating to demographic data and mental-health functioning (see details in the “Measures” section). Next, participants completed cognitive and behavioral tasks, which are not examined in the current study and are to be reported elsewhere. Following these tasks, a relaxation training session was conducted which utilized mindfulness-based relaxation techniques [42, 46]. Randomization was conducted by the project manager during the mindfulness training session to determine group placement of participant into one of two groups: (1) control group without a biofeedback device; (2) experimental group with biofeedback device. The first lab visit lasted approximately 65 min. Participants were also notified that a nightly survey would be sent at approximately 8 pm each night of the intervention. After 2 weeks, participants returned to the lab for their second visit, in which outcome data was collected via questionnaires.

If the participant was randomized into the experimental group, the research assistant would then conduct hands-on instruction with the participant on the use of the biofeedback device. This included information on how to properly pair the device to their phone, how to charge the device at night, and a high-level overview of the app’s functionality. After the participant’s device was properly paired and basic functionality of the device was understood and verified, the research assistant then provided the participant with the gift card and provided them information about daily surveys to be delivered each night. If the participant was placed in the control group, the research assistant would provide the participant with a gift card and ask them to fill out a daily survey that will be texted to them each night for the duration of the treatment. Participants returned to the laboratory after a 2-week interval. Participants received a monetary compensation of $40 ($20 pre-training and $20 post-training follow-up) for their time in the form of an Amazon gift-card. The same lab procedures were followed for the second lab visit. The local ethics review board approved the study. (Protocol reference: #IRB2015-0786D). This study was preregistered at ClinicalTrials.gov: NCT02837016. Registered 19 July 2016, https://clinicaltrials.gov/ct2/show/NCT02837016

### Mindfulness meditation training

Both arms received mindfulness meditation training. The mindfulness relaxation training script was designed by a clinical psychology graduate student and executed by trained undergraduate research assistants. In this training, the research assistant began by providing a brief overview of the theory behind mindfulness meditation and reviewed why it can work in controlling stress. First, the research assistants discussed how to breathe slowly and deeply for relaxation [47, 50] and monitored the participants breathing habits to ensure proper breathing techniques were established before moving on. Then, the research assistants discussed current theory and research on mindfulness meditation. After that, the research assistant left the room, while a 20-min mindfulness meditation audio was played on the computer. The audio emphasized awareness of the body and regulation of the breath [42, 46, 50]. Lastly the research assistant returned to the room and asked the participant if s/he has any follow-up questions. Additionally, the research assistant discussed how the participant might be able to integrate these techniques in their daily lives.

### Wearable respiratory-based biofeedback device

A biofeedback device was used that collects continuous respiration data and claims to provide real-time feedback through a messenger app when breathing patterns indicate tension. The device, which can sit comfortably on someone’s hip, will buzz and vibrate and send a message to the wearer’s phone when it detects erratic, defined by fast and highly variable, breathing patterns. The message may, for example, gently remind participants to mind their breath and do a deep breathing exercise to help alleviate stress. This device was chosen based on affordability, unobtrusiveness, and its focus on respiration.

Prior to data collection, the authors discussed the design and goal of the study with a senior member of the company. We received verbal confirmation we would be able to receive back end data, such as notifications per day, activity on the app, types of alerts received. This data could be anonymized based on device ID to allow for data matching. Further, we were notified that we would be able to also receive information about the specific algorithm used. After data was collected, initial findings were presented and shared with the senior member of the company.

### Measures

A scale was developed in order to gather information on possible covariates. The scale was administered after physiological hookup. Frequency of exercise was assessed using item ‘I exercise often,’ measured on a 5-point Likert scale ranging from strongly disagree to strongly agree. Whether participants were in treatment or taking medication were assessed using items, ‘Are you currently receiving any treatment for your stress?’ and ‘Are you prescribed any medications?” both measured through dichotomous, yes or no, responses. Alcohol usage was measured using item ‘How many days a week do you typically drink alcohol?’, with a scale between 1 and 7. Last, using the Macarthur Scale of subjective Social Status [74], individuals were asked to place themselves on a ladder based on their perceived social standing relative to their community on a scale between 1 and 10. Additionally, at the end of the lab visit, both groups were asked to answer questions on their motivation and expectation to change using items ‘How much do you expect a positive change?,’ and ‘How motivated are you to try new things to change?” on a scale from 1—very little to 10—very much.

#### State-Trait Anxiety Inventory (STAI)

The STAI is a self-report questionnaire that measures trait and state anxiety in adults [75, 76]. The term anxiety is used to describe both an unpleasant state (state-anxiety) as well as an individual’s relatively stable personality trait that describes an individual’s proneness towards anxiety (trait-anxiety) [76]. Please note that no clinical or diagnostic procedures were conducted which means findings may not generalize to individuals with anxiety disorders. Our findings can only speak to the symptomology associated with trait and state anxiety. For these reasons, we will adopt the more generic term ‘stress’ to describe the symptoms in this manuscript. The STAI is considered a widely used, reliable, and valid measure of stress and its symptomology [75–77].

#### Difficulties in Emotion Regulation Scale (DERS)

The DERS is a self-report questionnaire that measures multiple aspects of and difficulties with emotion regulation [78]. The DERS provides a total score as an indicator of emotion regulation difficulty, as well as subscales specific to emotion regulation strategies, non-acceptance of negative emotional responses, emotional clarity, impulse control, goal-directed behavior, and emotional awareness. This measure is a widely used and generally considered a reliable, and well-validated measure capturing difficulties in emotion regulation [78, 79].

#### Perceived Stress Scale (PSS)

The PSS is a self-report measure of how stressful one perceives his or her own life [80]. This scale was used as evidence indicates the importance and role of cognitive appraisal in perceptions of stress [81]. The PSS provides a single total score to reflect this stress level. This measure is a well-published, and generally considered a reliable and valid measure of perceived stress [80–82]. During conversion from paper to digital format, an error occurred causing item seven (‘In the last month, how often have you been able to control irritations in your life?’) to be left off from the questionnaire. Thus, this item is not reported or reflected within PSS pre-training or post-training scores.

#### Compliance

A nightly survey, for the entire 2-week intervention, was sent to both groups at 8 pm via text message through a service called Survey Signal [83]. This measure was used as an approximation for device-adherence. We asked participants to answer this survey as soon as they received it. The survey asked questions about whether a participant had meditated that day. A participant’s response, of either yes or no, was used as a measure of compliance (see Table 3). All continuously enrolled participants were sent both minimum and maximum number of fourteen surveys, one for each day they were enrolled in the study. Additional adherence was to be estimated based on the biofeedback company’s user data; however, the one-time data sent by the company resulted in a low match rate with our device identifiers and the company then became unresponsive despite multiple attempts to follow up.

### Analysis

SPSS version 24.0 [84] was used for the analyses. Repeated measures analysis of variance (RM-ANOVA) was used to compare the different treatment arms across time for each of the main outcome measures. For the main analyses, the STAI will be used to index changes in anxiety, the PSS will index changes in stress levels, whereas the DERS will be used to measure changes in the ability of emotion regulation. To assess group differences in age, sex, ethnicity, and grade type, chi-square and independent sample *t* test were used. Analyses of variances (ANOVAs) were used to test group differences in income, state-anxiety, and trait-anxiety. 95% confidence intervals (CI) are provided for the change-scores across session for each group (control and experimental) and group for each session time point (pre- and post-treatment).

No missing data existed on major variables (less than 5% for all measures) and no outliers were identified (scores above 2 standard deviations from the mean). Each individual was tested and included only once within our sample and analysis. Additionally, we have no reason to suspect that measurements for one subject may have influenced or is related to the measurements of other subjects and participants were explicitly instructed not to discuss the research study with anyone else.

## Results

The campus can be characterized as diverse, featuring students from urban and rural areas and different ethnic and socioeconomic backgrounds. Table 1 shows demographic data for the final sample broken down by group. The baseline values for age, gender, ethnicity, grade level, income, and state-anxiety and trait-anxiety levels did not differ among the groups.

**Table 1:** Descriptive statistics by group

	Control (***n*** = 18)	Experimental (***n*** = 21)	Total (***n*** = 39)
Age (years)	24.9 (7.5)	23.4 (3.6)	24.1 (5.7)
Sex			
Male	11 (61.1%)	10 (47.6%)	21 (53.8%)
Female	7 (38.9%)	11 (52.4%)	18 (46.2%)
Ethnicity			
White	7 (38.9%)	7 (33.3%)	14 (35.9%)
Black	–	2 (9.5%)	2 (5.1%)
Asian	7 (38.9%)	7 (33.3%)	14 (35.9%)
Hispanic	3 (16.7%)	4 (19%)	7 (17.9%)
Other	1 (5.6%)	1 (4.8%)	2 (5.1%)
Grade			
Undergraduate	8 (44.4%)	11 (57.1%)	19 (48.7%)
Graduate	10 (55.6%)	9 (42.9%)	20 (51.3%)
Income level			
< $34,999	9 (50.0%)	10 (47.6%)	19 (48.7%)
$35,000–$74,999	4 (22.2%)	6 (28.6%)	10 (25.6%)
$75,000 +	5 (27.8%)	5 (23.8%)	10 (25.6%)
State-Trait Anxiety Inventory			
State-anxiety			
Male	42.1 (5.3)	48.9 (12.2)	45.3 (9.7)
Female	45.9 (9.7)	48.8 (13.0)	47.7 (11.6)
Trait-anxiety			
Male	51.3 (4.6)	50.5 (7.8)	50.9 (6.2)
Female	51.2 (4.6)	54.0 (10.6)	53.5 (8.8)

*Notes.* Data are means (SD) or amount (%). Reported State-Trait Anxiety Inventory numbers are only at Pre-testing in order to assess group differences prior to intervention. No group differences were found on any variable

Additionally, the State-Trait Anxiety Inventory (STAI) [73] used to confirm high stress and anxiety levels in our sample through comparison with college-age normative scores. State-Anxiety refers to an unpleasant state, while trait-anxiety describes an individual’s proneness towards symptoms of anxiety [73]. Male and female participants scored in the 81st and 79th percentiles for state-anxiety, respectively, while both groups scored above the 90th percentile for trait-anxiety symptoms. Scores at or above the 75th percentiles, for both State and Trait-Anxiety, indicate clinically significant levels of anxiety that may affect their ability to function [73]. Again, chi-square and ANOVA results indicated no group differences among our sample (Table 1).

Preliminary analysis examined group differences on sociodemographic measures between group. See Table 2 for means and standard deviations for pre-training time and post-training time for both groups. No group differences on major descriptive variables were found. Eight additional items were examined: frequency of exercise, in treatment, taking medication, alcohol usage, social ladder perception, motivation to change, expectation to change, and compliance. Chi-square goodness of fit and *t* tests were used to assess group differences for each of the above variables. No significant group differences were found on all above variables (see Table 3) indicating no statistically significant group differences between the control versus experimental group on possible covariates including compliance.

**Table 2:** Means, standard deviations, and 95% paired means confidence intervals for dependent variables by group (control, experimental) and time (pre- and post-training)

	Control (***n*** = 18)	Experimental (***n*** = 21)	95% CI for MD
**State anxiety**			
Pre-training	43.6 (7.3)	48.9 (12.3)	− 5.3 95% CI (− 12, 1.4)
Post-training	38.6 (8.2)	43.4 (12.0)	− 4.8 95% CI (− 11.6, 2)
95% CI PMD	5 95%CI (− .1, 10)	5.5 95%CI (.8, 10.2)	
**Trait anxiety**			
Pre-training	51.8 (4.8)	52.3 (9.3)	− .5 95% CI (− 5.4, 4.4)
Post-training	47.3 (5.8)	49.6 (10.1)	− 3.3 95% CI (− 7.8, 3.2)
95% CI PMD	4.5 95% CI (2.3, 6.7)	2.7 95% CI (.67, 4.8)	
**Perceived Stress Scale**			
Pre-training	24.9 (4.8)	26.1 (3.5)	− 1.2 95% CI (− 3.9, 1.5)
Post-training	20.7 (5.7)	21.7 (5.8)	− 1 95% CI (− 4.7, 2.7)
95% CI PMD	4.2 95% CI (1.7, 6.8)	4.4 95% CI (2.1, 6.8)	
**Difficulties in emotion regulation**			
Pre-training	96.7 (22.0)	98.5 (21.6)	− 1.8 95% CI (− .16, 12.4)
Post-training	91.4 (18.3)	91.1 (18.1)	.3 95% CI (− 11.5, 12.1)
95% CI PMD	5.3 95% CI (− 2.8, 13.5)	7.4 95%CI (− .19, 15)	

*Notes.* Data are means (SD). No statistically significant differences between control and experimental group (all *p*’s > .05). *95% CI PMD* 95% CI Mean Difference

**Table 3:** Comparison of additional training related items between groups

		Control (***n*** = 18)	Experimental (***n*** = 21)	95% CI for MD
Frequency of exercise		2.9 (1.3)	3.3 (1.6)	− .4 95% CI (− 1.3, .5)
In-treatment				
Yes		2 (11.1%)	2 (9.5%)	–
No		16 (88.8%)	19 (90.5%)	–
Taking medication				
Yes		4 (22.2%)	2 (9.5%)	–
No		14 (77.7%)	19 (90.5%)	–
Alcohol usage		2 (1.4)	2.3 (1.6)	− .3 95% CI (− 1.3, .6)
Social ladder perception		6.1 (1.6)	6.2 (2.1)	− .1 95% CI (− 1.3, 1.1)
Motivation to change		7.6 (1.7)	7.9 (2.2)	− .3 95% CI (− 2, .5)
Expectation to change		7.2 (1.7)	6.9 (2.0)	− .3 95% CI (− 1.3, 1)
Compliance		9.3 (4.6)	8.1 (5.6)	1.2 95% CI (− 2.2, 4.5)

Notes: Data are means (SD) or amount (%). All *p*’s > .05. *95% CI PMD* 95% CI Mean Difference. Only pre-test numbers were assessed for covariate analysis. Frequency of exercise was measured on a 5-point Likert scale ranging from strongly agree to strongly disagree. Alcohol usage was measured by number of days per week. Social ladder perception, motivation to change, and expectation to change were measured in scores between 1 and 10. Compliance is the number of days participants answered the daily surveys

Our first objective regarding feasibility was to test compliance. While compliance was not statistically different between groups, the low average rate of compliance in both the control (*M* = 9.3, SD = 4.6) and experimental (*M* = 8.1, SD = 5.6) groups indicate our daily device-adherence measure to be insufficient without supplementary data on actual utilization. As for our secondary objective, the company failed to provide adequate supplementary data needed. We did not receive access to the raw data or gain  insight into the algorithms used to compute “stress level” from the breathing signal.

**Fig. 2:**
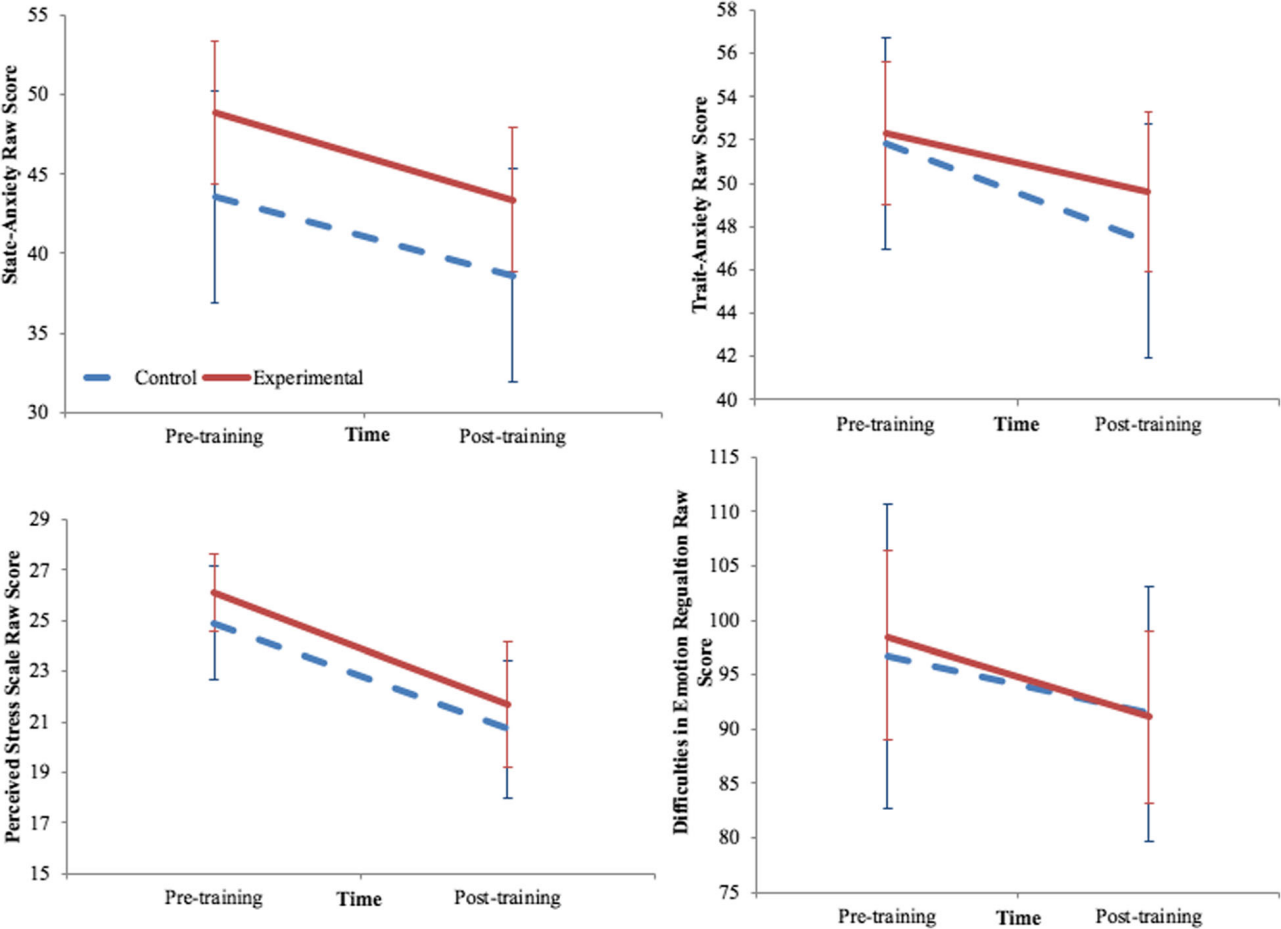
Scores for dependent variables state-anxiety, trait-anxiety, perceived stress, and difficulties in emotion regulation shown per group (experimental and control) and at both time points (pre- and post-training)

Figure 2 shows the data pre-training and post-training for our four outcome measures: the state anxiety, trait anxiety, perceived stress, and difficulty in emotion regulation.

To measure the effects of the wearable biofeedback device on state anxiety, trait anxiety, perceived stress, and emotion regulation, four separate 2 (group: experimental, control) × 2 (time: pre, post) RM-ANOVA with the outcomes measures as the dependent variable were conducted (Table 4). These analyses showed strong main effects of time for state anxiety (*F*(1,37) = 9.370, *p* = .004, η_p_^2^ = .202, trait anxiety (*F*(1,37) = 23.602, *p* = .000 , η_p_^2^ = .389, perceived stress (*F*(1,37) = 25.65, *p* = .000 , η_p_^2^ = .409, and emotion regulation (*F*(1,37) = 5.343, *p* = .026 , η_p_^2^ = .126. No main effects of group were identified for state anxiety (*F*(1,37) = 3.089, *p* = .087, trait anxiety (*F*(1,37) = .320, *p* = .575, perceived stress (*F*(1,37) = .65, *p* =.424, or emotion regulation (*F*(1,37) = .016, *p* = .901. No group × time interaction effect was statistically significant (all *p*’s > .237). Table 4 shows the relevant statistics and effects sizes from the RM-ANOVA.

**Table 4:** RM-ANOVA results for dependent variables

	df	***F*** value	***p*** value	η_p_^2^
State-anxiety				
Time**	(1,37)	9.37	0.004**	0.202
Group	(1,37)	3.09	0.087	0.077
Time × group	(1,37)	0.02	0.877	0.001
Trait-anxiety				
Time***	(1,37)	23.60	0.000***	0.389
Group	(1,37)	0.32	0.575	0.009
Time × group	(1,37)	1.45	0.237	0.038
Perceived Stress Scale				
Time**	(1,37)	25.65	0.000**	0.409
Group	(1,37)	0.65	0.424	0.017
Time × group	(1,37)	0.02	0.904	0.000
Difficulties in emotion regulation				
Time**	(1,37)	5.34	0.026**	0.126
Group	(1,37)	0.02	0.901	0.000
Time × group	(1,37)	0.14	0.712	0.004

*Notes.* Data are means (SD). *p ≤ .05. **p ≤ .01. ***p ≤ .001. Main effect for time was found for all dependent variables. No effect for group was found for dependent variables

## Discussion

### Data feasibility

Though participant compliance was not different between groups, the low average rate of compliance pointed towards a need for the biofeedback company’s actual utilization data to supplement our daily survey. However, due to lack of shared data regarding user behavior in relation to these devices and low transparency, we determine that the feasibility of such a study without full collaboration with private companies is low. The feasibility of future studies requires full collaboration with companies that includes detailed, agreed-upon procedures for data sharing. It is essential to ensure data from the back-end, such as verification that participants were in fact wearing the device, number of notifications provided by the company, as well as the user’s activity once a notification was received is provided in order to understand how this behavior interacts with other measures taken at the lab. Without this data, adherence can only be estimated and assumed. While we hoped that a randomized design would control for consumer behavior surrounding these devices, feasibility of the study was ultimately jeopardized. For future studies, we recommend formal written partnerships, as well as a detailed data passback policy to ensure transparency and successful data matching by participant. Additionally, future studies could design to accommodate for the low rate of participant adherence. For example, text messages could be sent as reminders on an intermittent basis. Furthermore, biofeedback devices that do not require frequent charging and that are waterproof may work best for younger samples so as to decrease actions needed for better adherence.

### Potential device effectiveness

Our secondary objective, examining whether this particular wearable respiratory-based biofeedback device would reduce stress more effectively than mindfulness meditation alone, ultimately showed no difference. Results of our small-scale trial showed that participants in the experimental biofeedback group were not different from the control group in their change of levels of stress before and after the treatment period. Our study did find that both the experimental and control group reported reduced levels of stress across the treatment period. As we did not utilize a proper control group to test for the effect of meditation training alone (e.g., a waitlist or active control group), it is possible that the beneficial effect could be due to the administration of the meditation training which was provided for both arms. These findings suggest that the wearable respiratory-biofeedback device did not have an added benefit in reducing stress above and beyond mindfulness meditation training and that the use of this specific device does not prove feasible for a larger scale study.

The current findings can be explained in several ways. First, it is possible that the device-algorithm was not working optimally. From a measurement perspective, accurately capturing respiratory information in real-life situations, in which participants are constantly moving and speaking, is challenging [85, 86]. Being able to link those signals back to specific emotions, among a possible myriad of other movements and states, may simply have been too complex. Although some basis for the wearable device was provided by the company [87, 88], these papers did not constitute an actual validation of the algorithms of the device. Unfortunately, we were unable to obtain the algorithms and, as such, the scientific contribution of our study is more utilitarian than mechanistic. Second, it is possible that the device was accurately capturing the respiratory signal but that the breath is simply not a good way to measure stress physiology in real-life situations. Currently, respiratory measurements are noted to be widely subjective depending on methodology, and previous studies have exposed reliability and validity issues relating to respiratory rate measurements [89]. Furthermore, to properly measure the breath, information on the rate, the volume, and the pattern must be reviewed in tandem. And while RSA and HRV are well validated in the literature, the introduction of new ways of measuring, such as apps or electrical equipment within cell phones, combined with possible artifacts generated through these new data captures, requires much more evidence to support the use of these devices for a clinical population. Third, these findings could indicate that the mindfulness meditation intervention paired with the breath regulation instruction alone may provide effective treatment, without the need for the use of a wearable biofeedback device.

Our results are difficult to situate in the current biofeedback literature. To the best of our knowledge, Gutierrez-Osuna’s group at Texas A&M University is only other group that conducted research utilizing a wearable biofeedback device capturing breathing rate as a means to influence stress. They showed that breath-based biofeedback training was more effective in inducing relaxation in participants than electrodermal- and cardiovascular-based biofeedback [90]. Though our studies are similar in some respects, they differ in that their study used the device as a training tool to remediate stress through a video-game interface, whereas in our study the device was used for the recognition of stress physiological markers.

### Limitations

Further limitations of the study should be considered in the development of a full-scaled study. One limitation is the lack of a waitlist control group (or active control group for the meditation alone) precluding statements regarding the effectiveness of the meditation. No waitlist control was utilized as we were primarily interested in the additive effects of the biofeedback device as opposed to the effects of meditation intervention per se. This study was planned as a phase 1 exploratory study testing the feasibility of a particular wearable biofeedback device that we intended to use in future, more detailed, studies to assess the general overall of the treatment compared to a waitlist or to determine the mechanism of action if results were positive.

Despite this limitation, we do point out that other studies which have utilized active control by meditation designs have found meditation interventions to generate positive effect sizes ranging from 0.30 to 0.67 [91, 92]. Also, previous studies conducted with students have found outcome variables relating to stress to either remain stable or increase in intensity between pre-test and post-test [85, 89]. As students in our study showed statistically significant decreases in stress measures, we believe, albeit speculatively, this could suggest that the meditation treatment was effective for high stress college students.

Furthermore, a few statistical limitations must be mentioned. We decided to run this study as a pilot to a future larger study as no previous studies have been conducted on our specific variables of interest and no effect size could be estimated. Because of this, the recruitment of highly stressed individuals combined with the smaller sample size may have affected the variability of our outcome variables reducing statistical power to detect meaningful findings. Additionally, it is also possible that regression to the mean, as well as test-retest effects could account for changes in both the test and control groups.

We also note that the present sample consisted of a non-clinical sample of college students who self-reported experiencing high levels of stress. It is possible findings could be different if a clinical sample was targeted in which people had confirmed diagnoses. Future clinical trials with diagnosed patients suffering from stress could help confirm the present findings.

Finally, we cannot exclude the possibility that the device was working properly but that the participants were simply not adhering to, or misunderstood, the instructions. We do believe this is unlikely as participants received more information on the use of the device than if they bought the device from the store. Furthermore, our team also made sure to demonstrate how to properly wear and use the device. Given the aforementioned limitations, it is important that future studies rigorously evaluate the validity of wearable biofeedback devices against currently existing, well-validated treatment options.

### Implications

To the best of our knowledge, this study is the first of its kind in studying a wearable respiratory-based biofeedback device in conjunction with mindfulness meditation through an RCT design. Strengths of this study include the application of these treatment options within an ecologically valid setting and the rigorous design using an active control group. Our findings suggest the biofeedback device provided no additive benefit to mindfulness training for stressed individuals which could be concerning as individuals with high stress levels seeking clinical solutions may be more susceptible to marketing messages. Furthermore, the authors caution against the use of commercially produced wearable biofeedback devices without Food and Drug Administration (FDA) approval for use in clinical settings and for patient monitoring. However, ultimately, as various limitations must be considered, future testing is recommended. Finally, we decided not to continue with the present company due to challenges related to transparency and information sharing.

## Conclusions

The goal of this study was to test the feasibility of using the biofeedback device, a respiration-based wearable biofeedback device claiming to reduce stress in a sample of college students. As such, our study is among the first to independently test commercially available biometric wearables. Our findings showed no added effectiveness of the specific respiratory-based biofeedback device we tested in reducing stress. Thus, we believe that these companies should comply with the same FDA standards required for medical companies. As it stands now, the lack of regulation can lead to public confusion and false hope in health-related industries. Furthermore, the validation of a commercial product proved difficult due to limited access to participants' behavior and adherence data regarding the device from the device maker. We assess that the feasibility of such a study proves difficult without closer collaborations between independent scientists and corporation in the research and development of such devices. With this, we would like to advocate for the collaboration of the FDA, academics, and private companies in the development of evidence-based regulations that will allow for a successful certification process that can bolster technological innovation while maintaining product quality, safety, and effectiveness. Ultimately, academia’s role as research and learning institutions can be strengthened through the government’s data collection and regulatory process capabilities, while industry can help close the gap between research and application through the development of products. Moreover, the open science research paradigm [93] can promote data sharing, preregistration, and funding source databases, and foster trust and collaboration across industries and disciplines.

## Supplementary Information

**Additional file 1.** CONSORT extension for Pilot and Feasibility Trials Checklist-Stress RCT Pilot.doc CONSORT Extension Checklist

## Data Availability

The datasets generated and/or analyzed during the current study are available in the Open Science Framework repository, https://osf.io/hec7a/

## Abbreviations

ANOVA: Analysis of variance
CBT: Cognitive behavioral therapy
DERS: Difficulties in Emotion Regulation Scale
FDA: Food and Drug Administration
HRV: Heart rate variability
PSS: Perceived Stress Scale
RCT: Randomized control trial
RM–ANOVA: Repeated measures analysis of variance
RSA: Respiratory sinus arrhythmia
STAI: State-Trait Anxiety Inventory
